# Facilitating access to health research through a participatory research register: a feasibility study in outpatient clinics

**DOI:** 10.1186/s40814-017-0148-5

**Published:** 2017-06-21

**Authors:** Verity A. Leach, John D. McGeagh, Ruta Margelyte, Niamh M. Redmond, Axel Walther, Sabi Redwood, Richard M. Martin, Jenny L. Donovan

**Affiliations:** 1 NIHR CLAHRC West, University Hospitals Bristol NHS Foundation Trust, Whitefriars, Bristol, BS1 2NT UK; 2 National Institute for Health Research Bristol Nutrition Biomedical Research Unit, Bristol, UK; 3 University Hospitals Bristol NHS Foundation Trust, Bristol, UK; 4 School of Social and Community Medicine, University of Bristol, Bristol, UK; 5 Department of Genetic Medicine, St Mary’s Hospital, Oxford Road, Manchester, M13 9WL UK

**Keywords:** Research participation, Prospective consent, Research registers, Research database, Research governance, Data linkage

## Abstract

**Background:**

A research register (Reach West) has been established to facilitate recruitment of people and patients to health-related research. We conducted a prospective feasibility study to investigate the practicality of recruiting through outpatient clinics.

**Methods:**

Patients over 18 years of age attending dental, eye or oncology outpatient clinics in an acute hospital in the West of England were provided with the opportunity to participate in Reach West. In Phase I, recruitment packs were handed to clinic attendees who could place completed consent forms in secure drop-box or return them later on-line or by post. In Phase II, recruitment packs were posted directly to patients with consent forms to be returned by post or on-line. Response rates by age, sex, postcode (for level of deprivation), and clinic type were recorded for those agreeing to participate on paper or on-line.

**Results:**

In Phase I, 2,314 of 4,500 (51.4%) of recruitment packs were handed out to clinic attendees, and 114 (5%) consented to join Reach West. In Phase II, 7,173 of 9000 packs were posted (79.7%), and 387 (5.4%) consented to participate. The overall consent rate was 6% (580), with the majority doing so on paper (87%) rather than on-line. The sample was balanced by sex, but mostly comprised people over 50 years located in less deprived postcodes. Non-staff costs for postal recruitment were lower than hand-outs in clinic (£6.84 compared with £8.05 per participant).

**Conclusions:**

Recruiting participants to the Reach West register was feasible among those with oncology, dental or eye outpatient appointments by post or with packs given out in the clinic. Response rates were similar to those achieved for other registers. Recruitment of participants can be achieved through outpatient clinics but other strategies will also be required to attract large numbers of participants and more diverse populations.

## Background

There are many research registers within the UK and beyond providing opportunities for members of the public and patients to take part in research, and facilitating access to potential study participants by researchers. Most of these registers are disease-specific or target a certain medical area [1–3]. However, in recent years, there is an increased interest in the use of universal research registers, where individuals prospectively consent to allow researchers to re-contact them to take part in future research opportunities [4]. These large registries, such as The Scottish Health Research Register (SHARE) [5] in Scotland, ResearchMatch [6] and the Women’s Health Register [7] (both the USA) have been successful in increasing research participation. Recruitment to research and patient and public involvement in research are high priorities for the UK’s National Institute for Health Research (NIHR) and National Health Service (NHS) [8]. However, the identification and recruitment of eligible participants into studies requires a large time commitment and staff resources [9] and is known to be challenging [10].

‘Reach West’ (https://reachwest.org.uk) has been developed as an online general research register open to all patients and the public over 18 years of age in the West of England, with the overarching aim of facilitating recruitment to approved health-related research studies projects [11]. Patients and members of the public who consent to take part in Reach West give consent for data linkage so that their NHS records and sociodemographic details can be used to identify research projects for which they might be eligible. Reach West participants can then be approached to take part in approved research studies, following usual ethical and governance processes [11]. Reach West has been approved by the South Central–Oxford C Research Ethics Committee (14/SC/1144). SHARE [5] is a similar register in Scotland, and while similar initiatives are being discussed elsewhere, to our knowledge Reach West is the first such register in England.

In this paper, we report on a study aiming to investigate the feasibility of recruiting to Reach West through routine outpatient clinics in England. The objectives were to assess the acceptability and practical aspects of recruiting in this way, and to evaluate response rates and basic costs.

## Methods

### Design

This prospective feasibility study was undertaken in routine adult oncology, dental and eye outpatient clinics in a single acute hospital in the West of England. There were two phases to investigate two recruitment strategies over a period of 13 weeks (7 March 2016 to 7 June 2016).

### Phase 1

Reception staff at the three outpatient clinics handed a participant information booklet (PIB), consent form and pre-paid envelope to all patients, their friends and family on arrival to reception at the clinic for a period of 5 days. There were no exclusion criteria for patients and their family/friends over 18 years of age. Completed consent forms were sent back to Reach West using the pre-paid envelope, on-line, or placed in a secure drop-box for returns placed at each clinic reception.

### Phase 2

The PIB, consent form and pre-paid envelope were included with consecutive outpatient appointment letters sent via the hospital’s usual external mailing company. The company was provided with 3000 packs per clinic and ran the mail-out until all packs had been sent for each clinic.

### Returned consent forms

Paper-based consent forms were labelled with a unique code (letter and number) for each clinic and recruitment method to enable tracking to the recruitment phase and particular clinics. It was not possible to track on-line consent forms. The paper consent forms received via the outpatient clinics were entered onto the Reach West secure database. Participants could also register directly on-line via the website as indicated in the PIB.

Where consent forms were returned with missing information or not fully completed, individuals were contacted to seek clarification of their intentions and to support them to complete the process. Those who consented under the age of 18 years were sent a letter with their returned consent form. All processes and outcomes were documented and filed for audit purposes.

### Analysis

The total number of packs handed out at clinics and via the external mail company were recorded allowing overall invitations and recruitment rates to be calculated. The consent form coding enabled the tracking and monitoring of recruitment rates and descriptive statistics were performed on these data. On-line consent was also recorded, but as these could not be traced to a particular clinic or specific recruitment phase, a combined recruitment rate was calculated. Basic socio-demographic information (date of birth and gender) was provided by participants. Home postcode data were used to calculate indices of multiple deprivation (IMD) to investigate response by levels of material deprivation [12].

Chi square statistics were used to compare gender, IMD score (high or low deprivation, 1–5 or 6–10) and age between on-line and paper consent. Three age groups were used because of small numbers aged under 50 or over 70 years.

A simple cost analysis for both recruitment methods was calculated by identifying the basic costs for each recruitment phase and applying the recruitment rate for each method to that cost. Costs included printing, stationary, and mail out company costs. Staff time was not included.

## Results

### Recruitment results

**Fig. 1:**
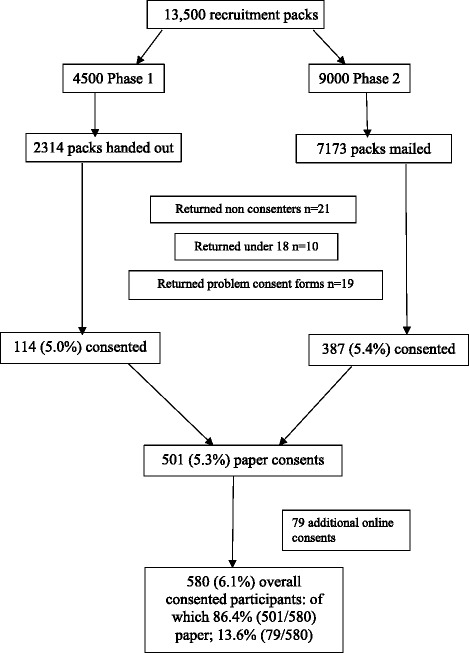
Participant invitation and flow chart

In total, 9487 recruitment packs were handed or mailed to patients in three outpatient clinics. Figure 1 details the flow of invitations and participants throughout the study. A total of 501 (86.4%) participants consented using the paper consent forms and 79 (13.6%) consented on-line (Table 1). This resulted in a total of 580 (6.1%) individuals consenting to participate in Reach West. In phase 1 (clinic hand out), 114 (5.0% of packs handed out) patients consented, with variation across clinics (Table 2), and in phase 2 (mail out), 387 (5.4% of packs mailed out) patients consented (Table 3).

**Table 1:** Total recruitment numbers across both phases

Phase of recruitment	Total recruited in that phase^a^	Total percentage (%) of both recruitment phases (*n* = 501)
1	114	22.6
2	387	77.4
Both	501	100

^a^Excludes participants joining on-line

**Table 2:** Reception staff hand out recruitment rates

Clinic	Total number of packs provided to clinic	Total number of packs handed out	No. of recruited patients (% of packs given out)^a^
Dental	1500	396	12 (3.0)
Eye	2000	1459	92 (6.3)
Oncology	1000	459	10 (2.2)
All clinics	4500	2314	114 (5.0)

^a^Excludes participants joining on-line

**Table 3:** Outpatient letter mail out recruitment rates

Clinic	Total number of packs provided to mail company	Total number of packs mailed out	No of recruited patients (% of packs mailed out)^a^
Dental	3000	2839	82 (2.9)
Eye	3000	2594	163 (6.3)
Oncology	3000	1740	142 (8.2)
All clinics	9000	7173	387 (5.4)

^a^Excludes participants joining on-line

Some consent forms were returned by people indicating that they did not wish to join Reach West, (*n* = 21) and 19 others were incomplete, with boxes unticked or forms unsigned. Ten under 18 years’ old completed the consent forms, however due they were ineligible.

### Participant demographics

Table 4 presents the demographics of the Reach West participants who consented. The participants were evenly split on gender (50.9% male). The largest age group was 50–69 years (44.3%), closely followed by 70+ (40.3%), with few under 49 year olds (15.3%). The majority of Reach West participants were mostly in the least deprived postcodes, with 70.6% of participants being in IMD decile 6 or above (least deprived scores).

**Table 4:** Feasibility study participant demographics

Demographic	On-line *n* (%)	Paper *n* (%)	Total *n* (%)
Gender			
Male	47 (54.5)	248 (49.5)	295 (50.9)
Female	32 (40.5)	253 (50.5)	285 (49.1)
Age			
70+	25 (31.6)	209 (41.7)	234 (40.3)
50-69	42 (53.2)	215 (42.9)	257 (44.3)
49 and under	12 (15.2)	77 (15.4)	89 (15.3)
IMD 2015 decile (LSOA 2011)			
1 (most deprived)	1 (1.3)	19 (3.8)	20 (3.4)
2	1 (1.3)	20 (4.0)	21 (3.6)
3	4 (5.1)	26 (5.2)	30 (5.2)
4	5 (6.3)	28 (5.6)	33 (5.7)
5	3 (3.8)	37 (7.4)	40 (6.9)
6	9 (11.4)	56 (11.2)	65 (11.2)
7	11 (13.9)	60 (12.0)	71 (12.2)
8	9 (11.4)	70 (14.0)	79 (13.6)
9	14 (17.7)	76 (15.2)	90 (15.5)
10 (least deprived)	21 (26.6)	84 (16.8)	105 (18.1)
Postcode unmatched	1 (1.3)	10 (2.0)	11 (1.9)
Postcode not provided	0 (0.0)	10 (2.0)	10 (1.7)

More males (59.5% of total on-line consenters) consented on-line compared with an equal divide in the percentage of male and female consenting on paper. A greater proportion of 50–69 year olds consented on-line (53.2%) compared to paper (42.9%), whereas a greater proportion of over 70 years olds opted to consent via paper (41.7%) compared to on-line (31.6%). There was no evidence of a difference between consent methods for 49 years and under olds (chi squared test, *p* = 0.963). There was some evidence of a difference in IMD decile scores between on-line and paper consenters for IMD score 6 and above (chi-squared test, *p* = 0.031). Eighty-one percent of on-line consenters were in IMD decile 6 or above compared to 69.1% of paper consenters. Table 5 shows comparisons of proportions for each demographic discussed.

**Table 5:** Demographic comparison of proportions

Demographic	Consent method (%)		Difference (%)	Confidence interval (95%)	Chi-squared^a^	*P* value
	Paper	Online				
IMD 6 or above	69.1	81.0	11.9	0.76–20.92	4.7	0.031
Gender - male	49.5	59.5	10.0	−2.47–21.8	2.7	0.099
50–69 years	42.9	53.2	10.3	−2.10–22.4	2.9	0.087
70+ years	41.7	31.6	10.1	−2.14–21.1	2.9	0.089
49 and under	15.2	15.4	0.2	−7.73–10.53	0.002	0.963

^a^Degrees of freedom = 1

### Comparison of costs

A simple comparison was made of costs between the two methods (Tables 6 and 7), showing that mailing out was less costly than hand-outs in clinic (£6.84 compared with £8.05 per participant (or £8.00 compared with £12.75 when all packs, used or not, was included). These costs did not include staff time as this was not documented separately from developmental and other tasks.

**Table 6:** Phase 1 costings

Item	Cost	Cost per participant recruited (*n* = 114)
Drop boxes (available only in clinics)	£176.49	£176.49
*Total packs printing* (*n = 4500*)	*£1,101.84*	n/a
Printing of packs handed out (*n* = 2314)	£566.59	£566.59
Printing of packs wasted (*n* = 2186)	£535.24	n/a
Stationary	£175.40	£175.40
Total	£1,453.73	£918.48
Cost per participant recruited (*n* = 114)	£12.75	£8.05

**Table 7:** Phase 2 costings

Item	Cost	Cost per participant recruited (*n* = 387)
Mail out costs (company)	£542.58	£542.58
*Total packs printing* (*n = 9000*)	*£2,203.69*	n/a
Printing of packs mailed (*n* = 7173)	£1756.34	£1756.34
Printing of packs wasted	£447.34	n/a
Stationary	£350.80	£350.80
Total	£3,097.07	£2649.72
Cost per participant recruited (*n* = 387)	£8.00	£6.84

## Discussion

To our knowledge, this is the first feasibility study assessing the practicality of recruitment via outpatient clinics to a general research registry. The overall consent rate was 6.1%. We found that recruitment by post with appointments or from information handed out in the clinic yielded similar numbers of participants overall. There was little difference in participation by gender, although slightly more males consented on-line. Older people (50–69 years or over 70 years) were more likely to consent to Reach West than younger (49 years and younger), probably reflecting the population attending these clinics. Mailing out reached more participants and incurred lower basic non-staff costs than handing out materials in clinics, and so would be more likely to be used in future.

It is difficult to compare our recruitment rate of 6.1% with other research registers. Green et al. (2013) [13] found recruitment rates to the CONNECT minority registry varied widely between 1 and 44%, depending on recruitment method. Harris et al. (2005) [14] found that less than 1% of the total subjects in the Vanderbilt Research program registered by paper, which was very different from Reach West. However, registers utilise a wide range of recruitment strategies, will vary in terms of numbers of eligible participants or locality. Further, many registers do not record baseline number of contacts. Registers that are similar, such as ResearchMatch and SHARE, run an open recruitment method rather than targeting specific population groups [6]. Specific registers such as for neurofibromatosis have shown the paid advertising was the most effective strategy, followed by referral by healthcare professional [15]. The recruitment rate of 6.1% for Reach West suggests it is feasible to recruit in this way, bearing in mind that this was a simple handing or mailing out of information in outpatient clinics.

The low rate of recruitment through the on-line process was unexpected in that other registers such as SHARE and ResearchMatch are primarily or completely completed on-line. However, the participants recruited to Reach West were mostly in older age groups, and so in future approaches to younger people would need to be explored.

### Strengths and weaknesses

The main strength of this study was the smooth running of the recruitment phases with tracking of the two recruitment methods. Sending material via the mail-out company, which was already working within the hospital, was straightforward and more reliable than handing out information packs. The study showed that recruitment to Reach West in hospital outpatient settings is feasible, although with several limitations. The study was small and based in three outpatient clinics in one acute hospital, and so the results may not be generalisable to other patient groups or settings. The outpatient clinics comprised mostly older people, and so there was limited participant diversity. The cost analysis was very basic with staff costs not included, and so it gives only a simple indicative cost. Materials were handed or mailed out without any formal endorsement or support from healthcare professionals, and it seems likely that more encouraging staff in one clinic (eye) did result in greater numbers of packs handed out and a slightly higher response.

### Future developments

As this study has shown that patients attending outpatient clinics will consent to participate in Reach West, albeit in small numbers, we now aim to plan further wider-ranging recruitment plans. In addition to endorsement or support from healthcare professionals, we will explore recruitment through other health-related organisations, and also local authorities, charities, or large employers. Social media is likely to be another option to facilitate large-scale recruitment, mostly on-line. It will be important to initiate targeted strategies to enhance recruitment of ‘hard to reach’ groups to increase participant diversity.

## Conclusions

In summary, the Reach West study showed that recruitment was feasible, with 580 (6.1%) of participants from three outpatient clinics consenting to join this general research register. However, more complex recruitment methods and marketing strategies will need to be implemented in future to increase participant numbers and diversity.
